# Complete genome sequence of *Pseudomonas brenneri* strain JD2-26, a potential pollutant-reducing bacterium isolated from a municipal solid waste landfill facility

**DOI:** 10.1128/mra.01094-24

**Published:** 2024-12-27

**Authors:** Kook-Il Han, Young Ho Nam, Hye Kyeong Kang, Hyun Mi Jin, Eui-Jin Kim

**Affiliations:** 1 Using Technology Development Department, Nakdonggang National Institute of Biological Resources (NNIBR), Sangju-si, Gyeongsangbuk-do, South Korea; 2 Microbiome Research Team, Food Science Center, Samyang Foods, Seoul, South Korea; University of Southern California, Los Angeles, California, USA

**Keywords:** *Pseudomonas brenneri*

## Abstract

We report here the complete genome sequence of *Pseudomonas brenneri* strain JD2-26 isolated from a municipal solid waste landfill facility. The genome consists of a 6.32-Mbp chromosome and a plasmid having a total of 6,100 genes, including 5,914 coding sequences.

## ANNOUNCEMENT

*Pseudomonas brenneri* belongs to the class Gammaproteobacteria, and its strains have been isolated from environments such as natural mineral waters (1), coalmine wastewater (2), contaminated soil (3), cold freshwater (4), and fish (5). In 2021, *Pseudomonas brenneri* JD2-26 was isolated from soil at a municipal solid waste landfill site (36°41′7″N, 128°26′96″E) in the Republic of Korea, using the dilution plating technique. Soil samples were collected in 50 mL sampling bags, diluted in 0.85% saline solution, and cultured on R2A agar (Difco, USA) at 30°C under aerobic conditions for 5 days. Single colonies were selected and purified through repeated streaking on R2A agar plates.

Strain JD2-26 was cultivated on R2A agar (Difco, USA) for 5 days at 30°C. Colonies were subsequently transferred with inoculation loops and suspended in the buffer provided with the Maxwell RSC Tissue DNA kit (Promega, USA) to isolate genomic DNA, following the manufacturer’s instructions. PacBio and Illumina sequencing were performed using the same DNA extract. For long-read sequencing, DNA was fragmented using a g-TUBE device (Covaris, USA), and a library was constructed with the 15-kb SMRTbell template preparation kit (Pacific Biosciences, USA). A size selection range of 7–12 kb was applied. After size selection, the SMRTbell library underwent annealing and binding according to the SMRT Link protocol, and sequencing was conducted on a Sequel II system. To improve the accuracy of the PacBio-generated contigs, additional Illumina sequencing was performed. A short-read library was prepared using the TruSeq Nano DNA kit (Illumina, USA) and sequenced on the Illumina NovaSeq platform, generating 151-bp paired-end reads. Library preparation and sequencing for both platforms were executed by Macrogen (Seoul, Republic of Korea). The PacBio Sequel II platform yielded a total of 102,820 reads (*N*_50_, 10,048 bp), while the Illumina platform produced 31,596,780 total reads. *de novo* assembly of the long reads was performed with HGAP4 software (6). Low-quality and short reads, along with adapter sequences and terminal bases, were filtered using Trimmomatic version 0.38 (7). Final error correction was carried out in three iterations using Pilon version 1.21 (8). The quality of the assembly was assessed using BUSCO version 5.1.3 (9) with the bacteria orthologs database v10. Gene prediction and initial annotation were conducted with Prokka version 1.14.6 (10). Circularity checking and overlap trimming were performed using SMRT Link (version 13.0.0.207600). As a result of *oriC* rotation, one circular chromosome and one circular plasmid were generated (Table 1). Coding sequence prediction and annotation were conducted using the NCBI Prokaryotic Genome Annotation Pipeline version 6.8 with GeneMarkS-2+ (11). Default parameters were used for all software otherwise specified.

**TABLE 1:** Summary of the assembly and annotation statistics for *Pseudomonas brenneri* JD2-26

Genetic element	Chromosome	Plasmid	Total
Genome size (bp)	6,321,865	231,733	6,553,598
GC content (%)	60	57.4	59.9
No. of coding sequences	5,679	235	5,914
No. of rRNAs	19	0	19
No. of tRNAs	69	0	69
Sequencing depth (×)	133.7	202.4	136.2
GenBank accession number	JBGRCL010000001	JBGRCL010000002	JBGRCL000000000

The genome consisted of a 6,321,865-bp circular chromosome and a 231,733-bp circular plasmid. The BLAST search revealed 99.19% sequence similarity between the plasmid and *Pseudomonas putida* plasmid pDK1 (AB434906.1). We identified multiple genes associated with pollutant degradation within the genome. The gene cluster included those responsible for the degradation of acetic acid and fatty acids (*accA*, *accB*, and *accC*), cresol (*bbsG*), hydrocarbons (*alkB*), biphenyl (*bphA* and *bphB*), catechol (*catA*, *catB*, and *catC*), dimethylphenol (*dmpA*), phenylalanine (*pheA*), and xylene (*xylA* and *xylB*).

## Data Availability

The genome sequence and raw sequencing reads for strain JD2-26 were deposited under GenBank accession number JBGRCL000000000, BioProject accession number PRJNA1151850, BioSample accession number SAMN43324894, and Sequence Read Archive (SRA) accession numbers SRR30802605 and SRR30802604. *Pseudomonas brenneri* strain JD2-26 has been deposited in the Korean Collection for Type Cultures under accession number KCTC 15888BP.
